# Stationary and Progressive Phenotypes Caused by the p.G90D Mutation in Rhodopsin Gene

**DOI:** 10.3390/ijms22042133

**Published:** 2021-02-21

**Authors:** Nina Kobal, Tjaša Krašovec, Maja Šuštar, Marija Volk, Borut Peterlin, Marko Hawlina, Ana Fakin

**Affiliations:** 1 Eye Hospital, University Medical Centre Ljubljana, Grablovičeva ulica 46, 1000 Ljubljana, Slovenia; nina.kobal.nk@gmail.com (N.K.); krasovec.tjasa@gmail.com (T.K.); sustar.majchi@gmail.com (M.Š.); marko.hawlina@gmail.com (M.H.); 2 Clinical Institute of Medical Genetics, University Medical Centre Ljubljana, Šlajmerjeva ulica 4, 1000 Ljubljana, Slovenia; marija.volk@kclj.si (M.V.); borut.peterlin@kclj.si (B.P.)

**Keywords:** rhodopsin, *RHO*, G90D, inherited retinal dystrophy, constitutively active mutation, retinal degeneration, retinitis pigmentosa, sector retinitis pigmentosa, pericentral retinitis pigmentosa, congenital stationary night blindness, RP, CSNB, fundus autofluorescence, FAF, electroretinography, ERG, OCT

## Abstract

Mutations in rhodopsin gene (*RHO*) are a frequent cause of retinitis pigmentosa (RP) and less often, congenital stationary night blindness (CSNB). Mutation p.G90D has previously been associated with CSNB based on the examination of one family. This study screened 60 patients. Out of these 60 patients, 32 were affected and a full characterization was conducted in 15 patients. We described the clinical characteristics of these 15 patients (12 male, median age 42 years, range 8–71) from three families including visual field (Campus Goldmann), fundus autofluorescence (FAF), optical coherence tomography (OCT) and electrophysiology. Phenotypes were classified into four categories: CSNB (*N* = 3, 20%) sector RP (*N* = 3, 20%), pericentral RP (*N* = 1, 6.7%) and classic RP (*N* = 8, 53.3% (8/15)). The phenotypes were not associated with family, sex or age (Kruskal–Wallis, *p* > 0.05), however, cystoid macular edema (CME) was observed only in one family. Among the subjects reporting nyctalopia, 69% (22/32) were male. The clinical characteristics of the largest p.G90D cohort so far showed a large frequency of progressive retinal degeneration with 53.3% developing RP, contrary to the previous report.

## 1. Introduction

Rhodopsin is the prototypical G protein-coupled receptor (GPCR) and a key light-sensitive protein of rod photoreceptors. During light activation, a photon is absorbed by its chromophore, 11-*cis*-retinal, which is covalently bound to Lys-296 via the protonated Schiff base (PSB). This is followed by a *cis*- to *trans*- photoisomerization of 11-*cis*-retinal that results in conformational changes in the opsin protein moiety, propagating the signal transduction by activating the protein transducin [1]. A key property of rhodopsin is its extremely high photosensitivity which enables single photon detection. This precision and high photosensitivity is achieved by remarkably low basal (constitutive) activity and highly efficient signal transduction and amplification [1]. Around 200 mutations in the rhodopsin gene (*RHO*) cause retinal diseases, in most patients in the form of retinitis pigmentosa (RP) [2,3,4,5], although other phenotypes have also been described, including sector RP [6], pericentral RP [7], congenital stationary night blindness (CSNB) [1,2,3,4,5], and retinitis punctata albescens [8]. Some of these pathogenic variants are thought to cause the photoreceptor to be constitutively active (activity in the absence of light) based on their conformational changes and the accessibility of the chromophore-binding pocket [3,9,10,11]. Constitutively active mutants have been associated with both RP and CSNB phenotypes [3,9,12,13,14]. The study, which was conducted in the United Kingdom, included 4236 individuals with inherited retinal disease from 3195 different families, observed the prevalence of *RHO* in 3,3% of individuals. However, *RHO* was one of the most frequently associated with autosomal dominant RP, along with *RP1* and *PRPF31* [15]. In another study, performed in Spain, which included 6089 individuals, the most frequent gene associated with autosomal dominant RP was *RHO* (31.4%) along with *PRPH2* (27%). Mutations in *PRPF31* were found in 16.4% individuals with autosomal dominant RP and *RP1* in 8% [16]. In our center, we treated 236 individuals with inherited retinal disease in the last two years. Among these, the proportion of individuals with the *RHO* mutation was 10.6% (25/236), of whom as many as 60% (15/25) had the p.G90D mutation and are included in our study. If we look only at the causative agents of the autosomal dominant form of RP, the most common causative agent was *RHO* (45.4% (25/55)) and the second most frequent was *PRPH2* (21.8% (12/55)).

The first (and only) family of seven patients with p.G90D was reported in 1995 by Sieving et al., who described a mostly stationary disease with the possible slight deterioration with age [17]. Since then, multiple biochemical studies focused on the characteristics of the mutation [3,9,12,13,14]. In vitro experiments suggest that the p.G90D belongs to the group of constitutively active mutants. The p.G90D mutant is able to activate transducin in the dark, resulting in a light-adapted state in the dark and the desensitization of rod photoreceptor cells [9]. The constitutively active G90D mutant also appears to adopt a conformation with impaired ability to bind arrestin [10].

We report the clinical characteristics of 15 patients from three Slovenian families, the largest p.G90D cohort so far, exhibiting four different phenotypes including a high frequency of progressive retinal degeneration, contrary to the previous report. Furthermore gender disbalance in the patient cohort was observed with males having an increased risk of disease.

## 2. Results

### 2.1. Clinical and Genetic Findings

**Figure 1:**
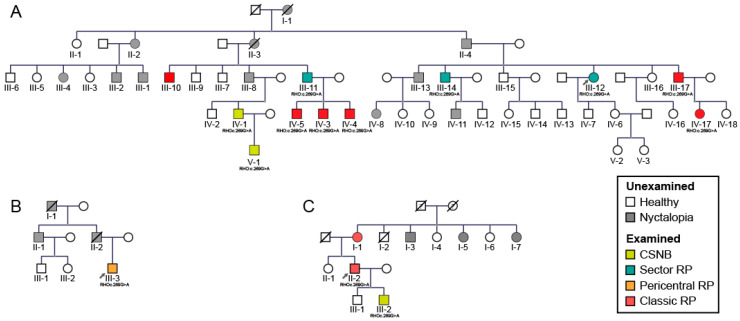
Pedigrees of three families (**A**–**C**) with the p.G90D mutation in rhodopsin gene *(RHO)*. Patient ID is shown underneath each symbol. Different phenotypes are shown in different colors. Unexamined affected family members are shown with grey symbols. Male subjects are indicated by squares and female subjects by circles. Arrows mark the index patient of each family which was diagnosed by whole exome sequencing while other examined patients had targeted sanger sequencing for the p.G90D variant. Roman numerals indicate generation while Arabic numerals indicate individuals in each generation. Abbreviations: CSNB—congenital stationary night blindness, RP—retinitis pigmentosa.

**Figure 2:**
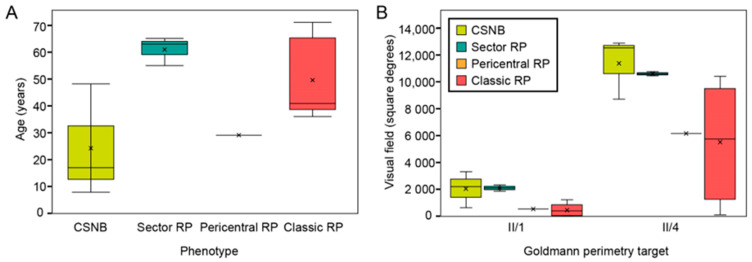
(**A**) Correlation between age and phenotype. (**B**) Patient visual field area in different phenotypic groups. Note that sector retinitis pigmentosa (RP) patients were of comparable or even older age than classic RP patients but had a better preservation of the visual field. The patients diagnosed with congenital stationary night blindness (CSNB) had normal visual fields but were relatively young thus it is possible that some visual field loss will occur with time. X-es indicate the average value in each phenotypic group. Abbreviations: CSNB—congenital stationary night blindness, RP—retinitis pigmentosa, II/1 and II/4—isopters II/1 (dim target) and II/4 (bright target) on Goldmann perimetry (described in detail in Methods).

This study screened 60 patients from three different families (as shown on pedigrees on Figure 1). Out of these 60 patients, 32 were affected and a full characterization was conducted in 15 patients. The clinical findings of these 15 patients with p.G90D mutation are summarized in Table 1. Genetic testing confirmed a mutation in p.G90D in the *RHO* gene in all (15) tested subjects. The patients were categorized into four phenotypes (CSNB, sector RP, pericentral RP and classic RP) predominantly based on the fundus autofluorescence (FAF) patterns and supported by other clinical findings (for details see Methods). Among the patients, 20% (3/15) were diagnosed with CSNB (all male, (median age 17 years; range 8–48); 20% (3/15) had sectoral RP (two male; median age 63 years; range 55–65), 6.7% (1/15) had pericentral RP (male, age 29 years) and 53.3% (8/15) of patients had classic RP (6 male, median age 42 years; range 36–71 years). The distribution of phenotypes among the three families is shown on the pedigrees on Figure 1 and the age distribution of each phenotypic group is shown in Figure 2. The distribution of phenotypes was not significantly associated with either family, age or sex (Kruskal–Wallis test, *p* > 0.05 for all) although there was a high frequency of males (86.7%, 13/15) in the studied cohort.

**Figure 3:**
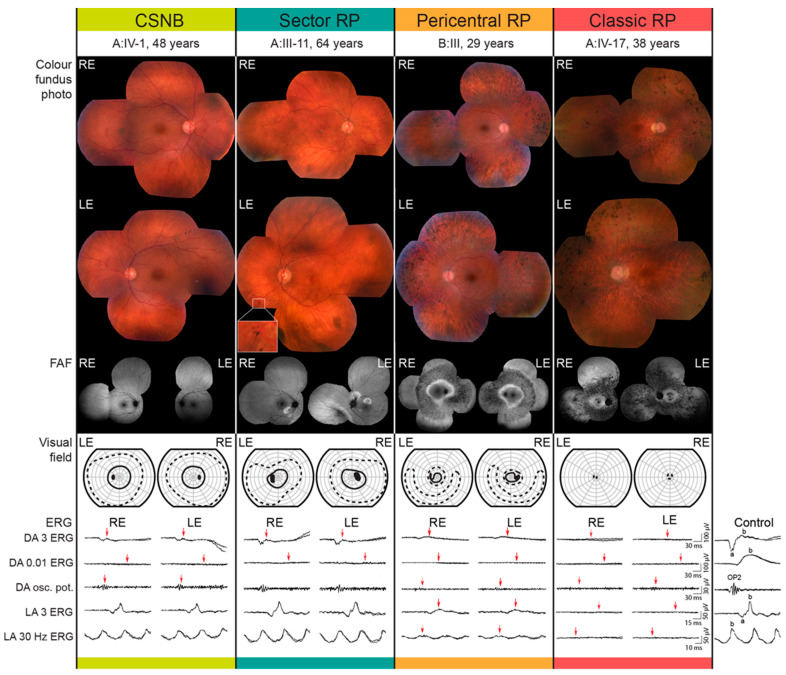
Representative cases from each phenotypic group. Color fundus photo, fundus autofluorescence (FAF), Goldmann visual field and full field electroretinography data are shown for representative patients from each phenotypic group. Different stimuli-recording protocol used for the full-field ERG recording is stated on the left and was chosen according to the standards and guidelines of the International Society for Clinical Electrophysiology of Vision (see Methods). Red arrows mark the deviation from the laboratory normatives. The ERG traces of a representative healthy control subject are shown on the right. Abbreviations: RE—right eye; LE—left eye; BCVA—best corrected visual acuity; ERG—electroretinography; DA—dark adapted; LA—light adapted.

**Figure 4:**
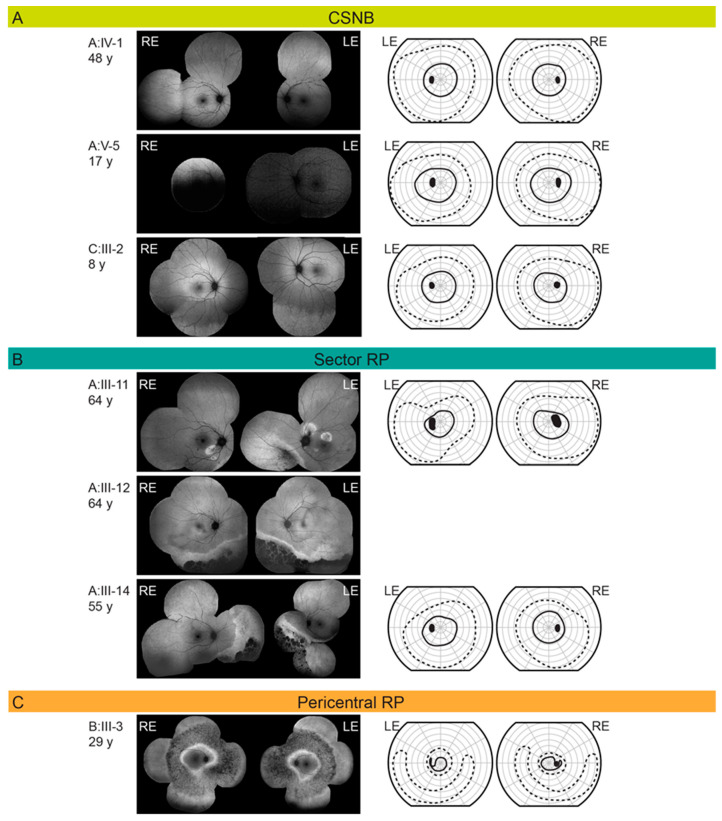
(**A**) Fundus autofluorescence and Goldmann visual field in *RHO* p.G90D patients diagnosed with congenital stationary night blindness (CSNB). (**B**) Fundus autofluorescence and Goldmann visual field in *RHO* p.G90D patients diagnosed with sector retinitis pigmentosa (RP). (**C**) Fundus autofluorescence and Goldmann visual field in *RHO* p.G90D patients diagnosed with pericentral retinitis pigmentosa (RP). Patient ID and age are stated on the left of each row. The full and dashed lines represent the II/1 (dim target) and II/4 (bright target) isopters, respectively. Abbreviations: CSNB—congenital stationary night blindness; RP—retinitis pigmentosa; RE—right eye; LE—left eye.

**Figure 5:**
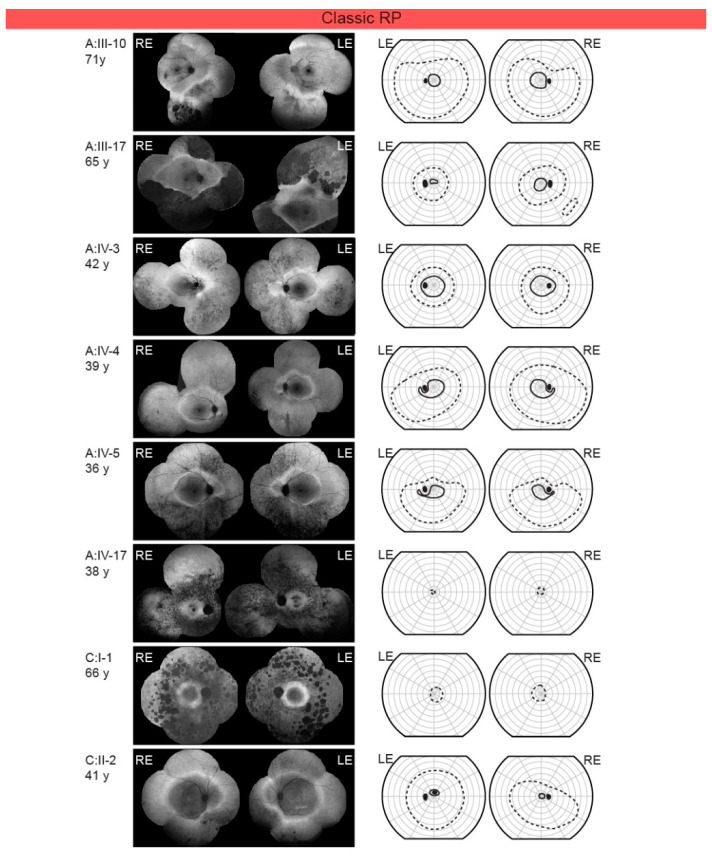
Fundus autofluorescence and Goldmann visual field in *RHO* p.G90D patients diagnosed with classic retinitis pigmentosa (RP). Patient ID and age are stated on the left of each row. The full and dashed lines represent the II/1 (dim target) and II/4 (bright target) isopters, respectively. Abbreviations: RP—retinitis pigmentosa; RE—right eye; LE—left eye.

All patients had night vision problems for as long as they could remember, which did not worsen with age. Four individuals (26.7%) reported daily visual problems with either central vision or visual field. First occurrences of daily visual problems were reported from as early as the second decade of life onward and the median age of onset was 42 (range 15–63) years. The median decimal Snellen best-corrected visual acuity for all tested eyes was 1.0 (range 0.1–1.0). Color vision was affected (≤9/15 Ishihara plates) in five patients, among whom four had classic RP (A:III-17, A:IV-17, C:I-1, C:II-2) and one sector RP (A:III-12). All patients diagnosed with CSNB were able to read all 15 Ishihara plates. Electroretinography (ERG) findings are summarized in Table 1 and representative responses for each phenotype are shown in Figure 3, together with the color fundus, visual field and FAF images. Figure 3, Figure 4 and Figure 5 show the visual field area in patients with different phenotypes. Visual field with II/1 isopter corresponded well with hyperautofluorescent borders on FAF (Figure 4 and Figure 5). Bone spicule pigmentation was seen in all individuals with RP, localized in different areas depending on the pattern of retinal degeneration (for examples see Figure 5), and none of the patients diagnosed with CSNB. FAF imaging revealed a normal pattern in two CSNB patients, whereas one CSNB patient (A:V-1) had a peripherally granular appearance which was thought to be within normal limits (Figure 4A).

Cystoid macular edema (CME) was present in 13.3% (2/15) of patients, all from the same family (C:I-1, C:II-2), both of whom had classic RP. In all, 10/15 patients had undergone ERG testing. The function of the rod system, as revealed by dark-adapted (DA) full-field ERG (ffERG), was highly dysfunctional in all patients. All CSNB patients and sector RP patient (A:III-11) and all patients with classic RP showed undetectable rod specific ERG (DA 0.01 ERG), while pericentral RP patient (B:III-3) had undetectable DA 0.01 ERG (Figure 3).The function of the cone system, as revealed by the light-adapted (LA) ffERG, was better preserved. One CSNB patient (C:III-2) had borderline and two (A:IV-1, A:V-1) had normal to slightly reduced LA responses with normal peak times and normal (A:V-1) and reduced (A:IV-1) S-cone response. Sector RP patient (A:III-11) had normal LA responses with undetectable S-cone response. Pericentral RP patient (B:III-3) showed reduced LA responses. Results in classic RP patients were as follows: one patient (A:III-10) had normal LA responses with prolonged peak times, one (A:III-17) had reduced LA responses and undetectable S-cone response and three (A:IV-17, C:I-1, C:II-2) had undetectable LA responses, two of whom (C:I-1, C:II-2) had undetectable S-cone response. Multifocal ERG (mfERG) revealed normal (A:V-1) or mildly reduced (A:IV-1) macular function in CSNB patients, normal macular function was also discovered in a sector RP patient (A:III-11), while the macular responses were reduced in the patient with pericentral RP (B:III-3). MfERG in classic RP patients revealed the following: one had mildly reduced macular function (A:III-10), one had reduced (A:III-17), one (A:IV-17) had undetectable responses in the macular region and one (C:II-2) had undetectable in the foveolar region and reduced towards periphery.

### 2.2. Gender Disbalance

Among the 60 subjects who were possible carriers of the mutation (marked with roman numerals on Figure 1), 57% (34/60) were male and 43% (26/60) were female (Table 2). Approximately half (53%, 32/60) of those were affected (marked with colored square/circle on Figure 1). Among those affected, there were 69% (22/32) male patients (Table 2). The higher risk for males was statistically significant, with males having approximately a three times higher risk of developing disease (logistic regression, Exp(B) = 2,9, *p* < 0.05). This analysis was then repeated using data from a previous study [17]: there were 65 potential carriers of the mutation, 54% (35/65) of whom were male and 46% (30/65) female (Table 3). Nyctalopia was reported in 52% (34/65) subjects, among whom 65% (22/34) were male (Table 3). There was a higher ratio of males among patients, however, this was not statistically significant (logistic regression, Exp(B) = 2.5; *p* = 0.07). However, when we pooled patients from our and their study (Table 4), we obtained a statistically significant difference with males having a 2.7 times (95% CI 1.3–5.6) higher risk of being affected (logistic regression, Exp(B) = 2.7; *p* < 0.01).

## 3. Discussion

In contrast to the previous report of p.G90D associated with CSNB-causing mutation [17], in the present study, only 20% (3/15) of patients had CSNB while others displayed various types of RP, with classic RP observed in 53.3% (8/15). Additionally, males were noted to have an approximately three times higher risk for developing disease than females.

### 3.1. Molecular Characteristics of RHO Mutations

The mutations in *RHO* have been previously categorized into two groups based on their effect on the rhodopsin molecule [5]. Group I includes mutations that are believed to affect rhodopsin synthesis, folding, or transport into the rod outer segments and are thought to affect the outer segment morphogenesis. One of the most frequent mutations in this group is P23H, the most common mutation linked with RP [5]. Group II mutations are not thought to affect the structural integrity of rhodopsin or outer segments; but rather affect rhodopsin’s functions, such as photobleaching, photoactivation, and deactivation. This group includes mutations that cause constitutively active forms of rhodopsin. Some of these are thought to cause only rod dysfunction (CSNB) (e.g., G90D, T94I, A292E, A295V) [1,2,4,9,10,18,19], while others were associated with retinal degeneration (RP) (e.g., G90V, S186W, D190N, K296E, K296M) [1,2,3,4,5,6,20]. The p.G90D mutation is located in the middle of the second transmembrane domain [21] and is thought to results in constitutive activity of the photoreceptor [1,3,6,9,12]. Biochemical studies have shown a blue-shifted absorbance maximum of G90D rhodopsin and a change in hydroxylamine accessibility, indirectly suggesting structural alterations in dark state rhodopsin that usually occur in light-activated wild-type rhodopsin [3]. The patients with this mutation were found to have a persistent loss of rod sensitivity [17], similar to that produced by continuous background, suggesting that the mutant rhodopsin stimulates the transduction cascade, producing an equivalent background light and light adaptation [3,22]. The desensitization in G90D patients was not reversed even after 12 h of dark adaptation [3,22]. All but one patient in the present study had absent dark-adapted rod responses on ERG, which is consistent with these observations. An exception was one patient with pericentral RP who had a residual rod response on ERG, suggesting possible variability in the degree of rod dysfunction; however, larger variability was seen in terms of retinal degeneration.

### 3.2. Mouse Models with RHO p.G90D

Several studies focused on mouse models of p.G90D [2,9,14,21,22,23,24]. The latest study by Colozo et al. observed no significant difference between the retina of wild-type (WT) and RhoTgG90D/+ mice (mouse heterozygous for the G90D rhodopsin mutant transgene on a heterozygous rhodopsin knockout background) at 6 months of age. Minimal differences were observed between the retina of WT and RhoTgG90D/TgG90D mice in young mice, where the rod outer segments in RhoTgG90D/TgG90D mice appeared to be shorter and the number of nuclei spanning the outer nuclear layer was lower. Progressive retinal degeneration was apparent as mice became older (6 months). This retinal degeneration was not caused by protein misfolding, as G90D rhodopsin was properly trafficked and localized to the outer segments of rod photoreceptor cells and Western blots of G90D rhodopsin from retinal extracts displayed a single band corresponding to a monomer of the receptor, indicating the absence of aggregates. Similar findings were reported by Dizhoor et al., which found no evidence of degeneration in heterozygous mice at any age but a small decrease in photoreceptor cell number in older animals. [22]. In the study, done by Sieving et al., transgenic mice harboring the G90D mutation had normal numbers of photoreceptors, but a considerable loss of rod sensitivity as measured by ERG. The desensitization effect increased with the copy number of mutant alleles, while it did not cause significant rod degeneration [14,24]. However, they also recognized that although retinas with transgenic opsin levels equivalent to one endogenous allele appeared normal for a period of approximately 3–4 months, retinal degeneration was observed in the late stages. Similarly, higher levels of G90D opsin expression produced earlier signs of retinal degeneration and more severe disruption of photoreceptor morphology [2,24]. However, the results were difficult to interpret as the rhodopsin was overexpressed in those mouse lines, which can cause retinal degeneration itself [9]. Based on the mouse studies, Conley and colleagues concluded that the G90D mutation manifests itself mostly as CSNB, but that degeneration can also occur later in life [23]. The heterozygous mice suffered from none or significantly milder retinal disease than our patient cohort, however, the reasons for this are unclear. Perhaps the degeneration occurs with years of continuing damage that is not possible to study in the short life span of mice. In fact, the youngest patient with any signs of degeneration was 29 years old and the median age of daytime visual symptoms (reflecting retinal degeneration) was 43 (range 15–63) years.

### 3.3. Genotype–Phenotype Correlations in RHO

Among almost 200 *RHO* mutations that have been described, most result in RP [1,6,20]. In addition to classic RP, milder forms have also been described, such as sector RP (e.g., Thr4Lys, Asn15Ser, Thr17Met, Pro23His, Thr58Arg and Gly106Arg) [6,25], pericentral RP (e.g., Gly18Asp, Gly51Arg, Thr58Arg, Gly106Arg and Cys187Phe) [7,26], and in one case retinitis punctata albescens (Arg135Trp) [8]. A small group of *RHO* mutations (G90D, T94I, A292E, A295V) [1,4,9,10,18,19], have been associated with CSNB, a non-progressive dysfunction of rod photoreceptors [1]. Topographically, mutations reported to cause CSNB were found to localize near the binding site for *cis*-retinal [4] and mutations causing sector RP, were predominantly found in the intradiscal domains [20]. Another mutation has been identified at the same amino acid as p.G90D—p.G90V. The p.G90V patients exhibited RP [13,19], and the authors postulated that the difference between the p.G90D (previously thought to cause only CSNB) and the p.G90V was in the ability to form hydrogen bonds with the opposing amino acids [19]. Considering the high frequency of RP in our p.G90D cohort, the two mutations were not as different as previously thought and possibly share the same pathogenic mechanism. Interestingly, another *RHO* mutation (p.E113K) has been described in association with both CSNB and RP in the same family [27]. It is possible that the genotype–phenotype correlations established for many *RHO* mutations are not completely resolved. First, there is usually a small number of examined patients for each mutation. Second, patients with mild phenotypes, such as CSNB, sector RP and pericentral RP often do not experience major visual problems and do not report to clinics. In fact, all three probands from this study had retinal degeneration (two severe) while relatives with milder phenotypes were only identified after a prospective invitation. The disadvantage of our study and study of Sieving et al. is that many affected patients were not examined. Perhaps RP phenotypes would be detected in the unexamined carriers from the previous study or unexamined patients in the current study would exhibit more CSNB phenotype. The other possibility is that other RP-associated mutation(s) dominate(s) the G90D mutation, resulting in a predominant RP phenotype.

Prospective studies are needed to establish definitive phenotypic spectrum of *RHO* mutations.

### 3.4. Phenotypes Associated with p.G90D

Patients in the present study exhibited four distinct phenotypes: CSNB, sector RP, pericentral RP and classic RP; the characteristics of each are described below.

#### 3.4.1. Congenital Stationary Night Blindness

CSNB caused by *RHO* mutations is of the Riggs type, electroretinographically characterized by the complete loss of rod-specific ERG activity. DA bright flash (3.0) ERG typically exhibits a reduced DA a-wave and low b-wave, while the LA cone-specific responses are largely normal, reflecting preserved cone function [28]. All three of our patients diagnosed with CSNB had typical electrophysiological features. CSNB is considered a non-progressive rod dysfunction without retinal degeneration [4,18,28]. The three older patients in the previously described p.G90D cohort exhibited some degeneration, however, the authors still diagnosed them with stationary disease [17]. On review, other *RHO*-CSNB mutations have not been associated with degeneration [3,4,14]. In comparison, achromatopsia is a congenital autosomal recessive cone dysfunction with a presumed stationary nature (e.g., GNAT2, ATF6…) [29,30], however, it has been suggested that it can sometimes show a predominantly stable genotype with a variable degree of retinal changes (e.g., CNGA, CNGB3…) [31,32] or genotype with a progressive loss of cone photoreceptors (e.g., PDE6C…) [33,34]. In parallel, the sector RP phenotype could potentially be the end-point of “CSNB” phenotype in p.G90D. Nevertheless, the high occurrence of degeneration in our p.G90D cohort warrants caution in diagnosing p.G90D patients with CSNB without a longer follow up into adulthood.

#### 3.4.2. Classic Retinitis Pigmentosa

RP is characterized by an initial loss of rod function, which is followed by the gradual loss of rod (and eventually cone) photoreceptor cells. The disease manifests with night blindness (nyctalopia) and the progressive loss of the peripheral visual field, resulting in “tunnel” vision, in some cases progressing to blindness [5]. Classic RP often begins with an annular scotoma similar to that in the pericentral RP; however the localization of the scotoma appears to be different. In classic RP, visual field defects initially appear in the midperiphery with an arcuate scotoma between 20°–40°. The restriction of the peripheral visual field appears early and becomes prominent, progressing to tunnel vision [35]. RP is also characterized by typical “bone-spicule” pigmentation and photoreceptor degeneration beginning in the mid-peripheral retina [26]. Patients often exhibit an increased autofluorescence ring which delineates the border between the affected and preserved retina [36]. All individuals with RP in our study had hyperautoflourescent rings. ERG findings in patients with RP are typically presented as the severe loss of both rod and cone-specific signals of peripheral retina [37]; however, milder or variable phenotypes have also been described as a feature of *RHO* mutations in patients with autosomal dominant RP [38]. In our subgroup of RP patients, 3/8 underwent ERG testing. Three had a complete loss of both cone and rod-specific signals (A:IV-17, F, 38y, Figure 3; C:I-1, F, 66y; C:II-2, M, 41y) and one had a severe functional abnormality of rod and milder abnormality of cone system (A:III-17; M, 65 y). Interestingly, one patient with RP (A:III-10; M, 71y) exhibited ERG abnormally that almost mimics Riggs type CSNB, with a well-preserved cone system response. However, a delayed peak time of cone responses indicated the initial deterioration of this system as well. He had a hyperautofluorescent ring on the FAF typical of RP and a constricted II/1 isopter (dim target); however, there was not much atrophy outside the ring and he had still relatively large II/4 isopter (bright target), suggesting the presence of residual cones outside of the ring.

#### 3.4.3. Sector Retinitis Pigmentosa

Sector RP is an uncharacteristic form of RP where only one or two quadrants of the retina are affected, in most cases inferior or nasal parts of the retina (superior visual field defects) possibly due to greater UV light exposure [25,39]. Sector RP has a favorable visual prognosis compared to generalized RP; it has been reported that 82% of cases will retain a visual acuity (VA) of 20/40 or better [39]. Generally, it is therefore considered a stationary to slowly progressive disease but may eventually lead to a more severe, diffuse RP phenotype [40]. All three patients with sector RP in our study had retinal degeneration in the inferior retina (Figure 4B) with concomitant superior visual field loss in the two with available visual fields (A:III-11 and A:III-14). Interestingly, the patient with sector RP who underwent ERG testing (A:III-11) showed the same ERG pattern as typically presented in non-progressive Riggs type of CSNB (Figure 3). The patient A-III:12 had worse visual acuity and color vision in comparison to the other two suggesting there is some variability also within this phenotype.

#### 3.4.4. Pericentral Retinitis Pigmentosa

Pericentral RP is an atypical form of RP that starts in the near periphery closer to the vascular arcades and tends to spare the far periphery. It is considered a milder form of RP [26]. In pericentral RP, visual field defects initially appear closer to the vascular arcades in comparison to classic RP; with an annular scotoma between 5°–30° and the preservation of the far periphery as the major distinctive features [41]. Patients with this subtype of RP usually exhibit an annular area of retinal degeneration encompassed by a double hyperautofluorescent ring [7,41]. This phenotype was observed in two patients in this cohort. Both had a typical FAF pattern, although the superior far peripheral visual field was affected in patient C:III-3 (Figure 4C). Pericentral RP presents with near-normal rod thresholds, while typical RP with rod reduced thresholds [41], although both of our patients with pericentral RP had reduced LA and DA responses, respectively. The pericentral RP patient had a preserved but reduced cone function on ERG and his rod function was undetectable (Figure 3).

Contrary to the previous report of mostly stationary rod dysfunction [1,2,3,4,5], RP was the most prevalent phenotype in this study, observed in 53.3% of patients. There was no statistical difference in age between the patients with classic RP, pericentral RP and sector RP (median ages of 42, 29, and 63 years, respectively), suggesting these phenotypes were not consecutive stages of one phenotype, but rather variants of different phenotypes associated with p.G90D. This holds especially for the pericentral RP and sector RP which affect spatially different areas of the retina. Classic RP could potentially be the end point of both, however the RP patients were not significantly older than those with sector and pericentral RP. The patients without signs of retinal degeneration were, however, relatively younger than other groups (median 17, range 8–48) and were therefore only cautiously diagnosed with CSNB, and it is possible that some retinal degeneration may occur with time. Considering that sector RP is also thought to be a stationary disease, it could potentially be the endpoint of the *RHO* “CSNB”. All sector RP patients were also older than CSNB patients, however, longitudinal studies are needed to confirm this. Patients III-10 and A:III-17 both have classic RP based on the FAF pattern of the hyperautofluorescent ring. The patient A:III-17 had greater degeneration outside the ring on FAF while cones on ERG had low amplitudes with normal latency, suggesting the normal function of the residual cones. On the other hand, patient A:III-10 had less degeneration on FAF and higher LA amplitudes, however, with prolonged peak times. We propose that patient A:III-10 probably has an active process in the peripheral retina, where degeneration is still occurring, whereas the degeneration process in the peripheral retina of pt. A:III-17 has reached a stable phase.

Only one study on seven patients from one family had previously reported a clinical phenotype associated with p.G90D [17]. All patients reported a non-progressive problem with night vision since childhood and the disorder was classified as CSNB. However, on careful review of the paper (Supplemental Table S1), 3/7 (43%) family members who were relatively older (age 38, 63 and 64) displayed some peripheral retinal degeneration. Those three were reported to have narrowed visual field and more abnormal ERG responses [17]. The study did not report FAF data and it is difficult to discern whether the phenotypes were more in keeping with sector RP or classic RP, nevertheless, the disease was not stationary in these patients. In comparison, the percentage of patients with retinal degeneration was still lower than in our study (81%), but there may be some interfamilial differences. Between our three families, the frequency of degeneration was 91% (10/11), 100% (1/1) and 75% (3/4). Furthermore, the youngest proband with degeneration in the cohort of Sieving et al. was 38 years old, while in our study, degeneration was observed as early as 17 years of age (the youngest patients with degeneration were 17, 29 and 41 years old in the three families). On review of other *RHO* mutations associated with CSNB (T94I, A292E, A295V), there were no reports of progressive disease [4,18,42,43].

### 3.5. Gender Disbalance

Among the 60 subjects who were possible carriers of the mutation (marked with roman numerals in Figure 1) approximately half (54%, 32/60) were affected, which is consistent with the dominant inheritance of *RHO* allele. However, among those affected, there were more male patients than expected (69%, 22/32) in comparison to relatively even gender distribution within the families. The higher risk for males was statistically significant with males having around three times higher risk of developing disease than females. Moreover, when adding data from another study on this mutation [17] the difference was even more significant. Such an observation has not yet been reported for *RHO* retinopathy and is unexpected for an autosomal disease. Interestingly, gender disbalance was recently also reported in ABCA4-retinopathy, an autosomal recessive disease—where there were significantly more females with mild alleles in the patient cohort [44]. Another study, performed on rd10 mice (a model of autosomal recessive RP), reported an earlier onset of and faster rate of rod degeneration in female mice [45].

It is not clear why there is such high clinical variability between the patients with the same mutation. Modifying genetic and external factors may affect disease expression and the identification of these could have a potential value in developing novel treatments. One of the factors could be gender and thus the existence x-linked genetic modifiers or hormonal influence is possible [45]. Interestingly, CME was seen only in patients from one family, suggesting that the susceptibility to this frequent complication of RP could have a genetic background.

## 4. Materials and Methods

### 4.1. Patients

The study involved 15 patients, three female and twelve male, with a median age of 42 in the range of 8–71 years from three families. One patient from each family (A:III-12, B:III-3 and C:I-1) was identified in the database of rare genetic eye diseases of University Eye Hospital Ljubljana. The other patients were recruited by inviting all family members who reported nyctalopia.

All investigations were carried out in accordance with the Helsinki Declaration on Biomedical Research in Human Beings. The study was approved by the Commission of the Republic of Slovenia for Medical Ethics (Protocol 0120-435/2020/3, 20 October 2020). Patients signed their informed consent.

### 4.2. Genetic and Bioinformatic Analysis

Genomic DNA was extracted from blood samples according to the standard procedure. Genetic analysis was performed by whole exome sequencing in one proband from each family (marked with arrows on the pedigrees, Figure 1). Sequencing of the defined targets was performed using next-generation sequencing on the isolated DNA sample in proband from each family (A:III-12, B:III-3 and C:I-1). Briefly, the fragmentation and enrichment of the isolated DNA sample were performed according to the Illumina Nextera Coding Exome capture protocol, with subsequent sequencing on Illumina NextSeq 550 in 2 × 100 cycles. After duplicates were removed, the alignment of reads to UCSC hg19 reference assembly was performed using the Burrows–Wheeler aligner (BWA) algorithm (v0.6.3) and variant calling was performed using the GATK framework (v2.8). Only variants exceeding the quality score of 30.0 and depth of 5 were used for down-stream analyses. Variant annotation was performed using ANNOVAR and snpEff algorithms, with pathogenicity predictions in the dbNSFPv2 database. Reference gene models and transcript sequences are based on RefSeq database. Structural variants were assessed using the CONIFER v0.2.2 algorithm. Variants with population frequency exceeding 1% in gnomAD, synonymous variants, intronic variants and variants outside the clinical target were filtered out during analyses. An in-house pipeline was used for the bioinformatic analyses of exome sequencing data, in accordance with the GATK best practice recommendations [46]. The interpretation of sequence variants was based on ACMG/AMP standards and guidelines [47]. When sequencing the DNA sample, we reached a median coverage of 67× and covered over 99.9% targeted regions with a minimum of 10× depth of coverage [48]. Mutation in other family members was confirmed using Sanger sequencing. Primers are available upon request.

### 4.3. Clinical Examination

Individuals were questioned considering the age of onset of their night vision problems and problems with daytime vision which included worse visual acuity and/or the narrowing of the visual field. Each patient underwent ophthalmological examination, which included Snellen visual acuity, color vision (Ishihara), slit lamp examination, visual field tests, color fundus photography (Topcon, Tokyo, Japan), FAF and OCT (Spectralis, Heidelberg Engineering, Dossenheim, Germany) and ERG. The color fundus and FAF images were combined into mosaics using the i2k Retina software (DualAlign LLC, Clifton Park, NY, USA). Visual field tests were performed using manual kinetic Goldmann perimetry. The stimuli II/1 and II/4 (size of 1 mm^2^; luminance of 10 and 318 cd/m^2^, respectively) were used. The area of the visual field for individual isopters was determined using imageJ sofware (NIH, Bethesda, MD, USA) after scanning the visual fields. ERG was performed according to the standards and guidelines of International Society for Clinical Electrophysiology of Vision [49,50,51], using Espion (Diagnosys LLC, Lowell, MA, USA) or RETI scan (Roland Consult Stasche & Finger GmbH, Germany) visual electrophysiology testing systems. FfERG was used to assess the general retinal function with the following recording protocols: dark-adapted 0.01 ERG (DA 0.01 ERG; response of the rod-driven on-bipolar cells), dark-adapted 3 ERG (DA 3 ERG; combined responses from photoreceptors and bipolar cells of both the rod and cone systems, but mostly rod dominated), dark-adapted oscillatory potentials (DA osc. pot.; responses mostly from amacrine cells), light-adapted 3 ERG (LA 3 ERG; responses of the cone system; a-waves originates from cone photoreceptors and cone off- bipolar cells, while the b-wave arises from on- and off-cone bipolar cells), light-adapted 30 Hz flicker ERG (LA 30 Hz; cone-pathway-driven response) [49]. S-cone ERG (the selective response from the S-cone system) was elicited with a 0.03 cd s/m^2^ blue (449 nm) stimuli on a bright (100 cd/m^2^) amber (594 nm) background. MfERG [50] and/or pattern ERG (PERG) [51] were used to assess the function of the macula. MfERG testing was performed with the stimuli of 60° in the diameter, presented on a cathode-ray tube monitor. The stimulus included an array of 61 hexagons, which were modulated between light (L) and dark (D) with 96–98% contrast according to a binary m-sequence (511 samples of the sequence: LDDDD). PERG was elicited with 0.8° checkerboard pattern, presented on a 21.6° × 27.8° CRT screen stimulator. The checkerboard pattern was reversing 1.8 times per second, and the contrast between black and while fields was 99%. The signals were amplified and stored in a hard disc on the computer for further analysis.

### 4.4. Phenotype Classification

Phenotypes were classified into four categories:

1. Congenital stationary night blindness (CSNB): normal FAF and visual field;
2. Classic retinitis pigmentosa (RP): concentric retinal degeneration delineated by a hyperautofluorescent ring on FAF;
3. Sector RP: peripheral degeneration extending one to two quadrants on FAF;
4. Pericentral RP: annular area of retinal degeneration encompassed by a double hyperautofluorescent ring on FAF.

### 4.5. Statistical Analysis

Statistical analysis was performed using SPSS software (ibm.com). Median values (age and visual field) between different phenotypic groups were compared using the Kruskal–Wallis test. Disease risk in association with different genders was determined by logistic regression.

## 5. Conclusions

Among 15 patients from three families, the largest *RHO* p.G90D cohort to date, only 20% had CSNB, a non-progressive rod dysfunction, previously associated with this mutation. On the contrary, 80% exhibited retinal degeneration, in the form of classic RP in half of the cases. Furthermore, males had an approximately three times increased risk of developing disease, a novel finding in *RHO* and unusual for an autosomally inherited mutation.

## Figures and Tables

**Table 1:** Results of ophthalmological examination, electroretinography and phenotype classification.

Patient ID	Sex	Age at Last Visit (Years)	Problems with Daytime Vision (Age at Onset)	BCVA (Snellen)		Color Vision (Ishihara Plates Read; ≤9/15 = Abnormal)		Visual Field—II/1 Isopter Field Area (deg^2^)		Bone Spicule Pigmentation	Fundus Autofluorescence Pattern	CME	ERG	Phenotype Classification
				RE	LE	RE	LE	RE	LE					
A:III-10	M	71	no	1.0	0.9	12/15	12/15	610	385	Yes	Hyperautofluorescent ring	No	BE: mfERG mildly reduced ffERG undetectable DA 0.01 ERG, normal LA amplitudes with prolong peak times	Classic RP
														
A:III-11	M	64	no	1.0	1.0	15/15	15/15	2045	1592	Yes	Sectoral degeneration	No	BE: mfERG normal, ffERG undetectable DA 0.01 ERG, normal LA responses, undetectable S-cone ERG	Sector RP
														
A:III-12	F	64	yes (63)	0.7	0.6	1/15	1/15	N/A	N/A	Yes	Sectoral degeneration	No	N/A	Sector RP
														
A:III-14	M	55	no	1.0	1.0	N/A	N/A	2298	2309	Yes	Sectoral degeneration	No	N/A	Sector RP
														
A:III-17	M	65	no	0.9	0.8	4/15	2/15	342	81	Yes	Hyperautofluorescent ring	No	BE: mfERG reduced, ffERG undetectable DA 0.01 ERG, reduced LA responses, undetectable S-cone ERG	Classic RP
														
A:IV-1	M	48	no	1.0	1.0	15/15	15/15	2073	2233	No	Normal	No	BE: mfER mildly reduced, ffERG undetectable DA 0.01 ERG, normal to slightly reduced LA responses with normal peak times, reduced S-cone ERG	CSNB
														
A:IV-3	M	42	no	0.9	1.0	15/15	15/15	1220	1147	Yes	Hyperautofluorescent ring	No	N/A	Classic RP
														
A:IV-4	M	39	no	1.0	1.0	15/15	15/15	876	927	Yes	Hyperautofluorescent ring	No	N/A	Classic RP
														
A:IV-5	M	36	no	1.0	1.0	15/15	15/15	537	553	Yes	Hyperautofluorescent ring	No	N/A	Classic RP
														
A:IV-17	F	38	yes (15)	0.6	0.4	1/15	1/15	0	0	Yes	Hyperautofluorescent ring	No	BE: mfERG undetectable, ffERG undetectable	Classic RP
														
A:V-1	M	17	no	1.0	1.0	15/15	15/15	3189	3377	no	Sectoral degeneration	No	BE: mfERG normal, ffERG undetectable DA 0.01 ERG, normal to slightly reduced LA amplitudes with normal peak times, normal S-cone ERG	CSNB
														
B:III-3	M	29	no	1.0	1.0	10/15	13/15	511	443	Yes	Double hyperautofluorescent ring	no	BE: mfERG reduced, ffERG reduced, DA 0.01 ERG, reduced LA amplitudes with prolonged peak times	Pericentral RP
														
C:I-1	F	66	yes (46)	0.1	0.1	1/15	1/15	0	0	Yes	Hyperautofluorescent ring	yes	BE: ffERG: undetectable DA 0.01 ERG, undetectable LA ERG amplitudes, undetectable S-cone ERG, PERG significantly reduced	Classic RP
														
C:II-2	M	41	yes (37)	0.2	0.1	0/15	0/15	91	107	Yes	Hyperautofluorescent ring	yes	BE: mfERG: undetectable in the foveolar region and reduced towards periphery; ffERG: undetectable DA 0.01 ERG, reduced LA ERG amplitudes with severely prolonged peak times, undetectable S-cone ERG	Classic RP
														
C:III-2	M	8	no	1.0	1.0	15/15	15/15	933	252	No	Normal	no	BE: ffERG: undetectable DA 0.01 ERG, borderline LA ERG amplitudes with borderline prolonged peak times	CSNB

Abbreviations: M—male, F—female; RE—right eye; LE—left eye; BE—both eyes; BCVA—best corrected visual acuity; CME—cystoid macular edema; ERG—electroretinography; mfERG—multifocal electroretinography; ffERG—full field electroretinography; DA—dark adapted; LA—light adapted, PERG—pattern electroretinography; N/A—not available; CSNB—congenital stationary night blindness; RP—retinitis pigmentosa. Fundus autofluorescence and electrophysiology results were highly symmetrical between the eyes.

**Table 2:** Gender disbalance in patients harboring p.G90D in *RHO* in our study.

	Potential Carriers	Affected	Unaffected
Male	34 (57%)	22 (69%)	12 (43%)
Female	26 (43%)	10 (31%)	16 (57%)
Total	60	32	28

In our study, there were 60 possible carriers of the mutation, 57% (34/60) were male and 43% (26/60) were female. Note a high percentage of affected males (69%, 22/32) in comparison to females (31%, 10/32).

**Table 3:** Gender disbalance in patients harboring p.G90D in *RHO* from previous study [17].

	Potential Carriers	Affected	Unaffected
Male	35 (54%)	22 (65%)	13 (42%)
Female	30 (46%)	12 (35%)	18 (58%)
Total	65	34	31

There were 65 possible carriers of the mutation, 54%% (35/65) were male and 46% (30/65) were female. Note a high percentage of affected males (65%, 22/34) in comparison to females (35%, 12/34).

**Table 4:** Gender disbalance in patients harboring p.G90D in *RHO* from this and previous study.

	Potential Carriers	Affected	Unaffected
Male	69 (55%)	44 (67%)	25 (42%)
Female	56 (45%)	22 (33%)	34 (58%)
Total	125	66	59

The Table 4 includes data from the present study and data gathered from the pedigree of the previous study [17]. Note a high percentage of affected males even though the genders were relatively evenly distributed among potential carriers.

## Data Availability

The original data are available upon reasonable request to the corresponding author.

## Supplementary Materials

The following are available online at https://www.mdpi.com/1422-0067/22/4/2133/s1, Table S1: Clinical details of the patients harboring *RHO* p.G90D gathered from a previously published report.

## Abbreviations

RHO	Rhodopsin gene
CSNB	Congenital stationary night blindness
RP	Retinitis pigmentosa
OCT	Optical coherence tomography
FAF	Fundus autofluorescence imaging
ERG	Electroretinography
GPCR	G protein-coupled receptor
PSB	Protonated Schiff base
CME	Cystoid macular edema
DA	Dark adapted
LA	Light adapted
WT	Wild type
UCSC	University of California Santa Cruz
BWA	Burrows–Wheeler aligner
GATK	Genome Analysis Toolkit
CONIFER	Copy Number Inference From Exome Reads
gnomAD	Genome Aggregation Database
ffERG	Full-field electroretinography
mfERG	Multifocal electroretinography
PERG	Pattern electroretinography
