# Supplementary material for: Stationary and Progressive Phenotypes Caused by the p.G90D Mutation in Rhodopsin Gene
Source: Int J Mol Sci. 2021 Feb 21;22(4):2133. doi: 10.3390/ijms22042133 (PMC7924842; doi:10.3390/ijms22042133)
Supplement: Supplementary file 1 [file ijms-22-02133-s001.pdf]

Table 1. Clinical details of the patients harboring *RHO* p.G90D gathered from a previously published report [1].

Patient ID	Sex	Age at the the time of the study (years)	BCVA (Snellen) BE	Color vision	Problems with vision	Fundus features	Visual Field (Goldmann perimetry)	ERG
IV-1	M	11	1.0	normal	non-progressive problems with night vision since childhood	normal	full	marked loss of rod ERG responses, tested by dim blue flashes in the dark adapted state, absence of a waves
III-12	F	31	1.0	normal	non-progressive problems with night vision since childhood	normal	full	marked loss of rod ERG responses, tested by dim blue flashes in the dark adapted state, absence of a waves
III-10	F	32	1.0	normal	non-progressive problems with night vision since childhood	normal	full	marked loss of rod ERG responses, tested by dim blue flashes in the dark adapted state, absence of a waves
III-9	F	33	1.0	normal	non-progressive problems with night vision since childhood	normal	full	marked loss of rod ERG responses, tested by dim blue flashes in the dark adapted state, absence of a waves
III-6	M	38	1.0	normal	non-progressive problems with night vision since childhood	pigment group formation in periphery	constricted	marked loss of rod ERG responses, tested by dim blue flashes in the dark adapted state, absence of a waves, absence of normal amplitude of cone response to 30Hz flicker
II-3	M	63	1.0	normal	non-progressive problems with night vision since childhood	some pigment group formations, minimal atrophy, minimally attenuated arterioles	minimally constricted	marked loss of rod ERG responses, tested by dim blue flashes in the dark adapted state, absence of a waves
II-2	M	64	1.0	normal	non-progressive problems with night vision since childhood	pigment group formation in periphery	constricted	marked loss of rod ERG responses, tested by dim blue flashes in the dark adapted state, absence of a waves, absence of normal amplitude of cone response to 30Hz flicker, absence of normal cone response to single flashes, light adopted, remaining small responses had normal waveform and timing, there were fewer cones but functioning well

Abbreviations: M- male, F – female, BCVA – best corrected visual acuity, BE – both eyes, ERG – Electroretinography

1. Sieving, P.A.; Richards, J.E.; Naarendorp, F.; Bingham, E.L.; Scott, K.; Alpern, M. Dark-light: model for nightblindness from the human rhodopsin Gly-90-->Asp mutation. *Proceedings of the National Academy of Sciences of the United States of America* **1995**, *92*, 880-884, doi:10.1073/pnas.92.3.880.
